# Supplementary material for: Functional beverage development from traditional Thai polyherbal tonic: Antioxidant-rich microcapsules and comprehensive sub-chronic toxicity assessment
Source: PLoS One. 2025 Dec 23;20(12):e0339571. doi: 10.1371/journal.pone.0339571 (PMC12725736; doi:10.1371/journal.pone.0339571)
Supplement: S1 Table — (DOCX) [file pone.0339571.s001.docx]

**Supplementary Table S1** Composition of *Phyllanthus emblica*-based functional herbal tea with varying proportions of herbs and spices (grams of each plant per 2.5 g).

**Scientific names Phy-Blica-O Phy-Blica-B Phy-Blica-D Phy-Blica-E**

*Aegle marmelos* (L.) Correa ex Roxb. - - 0.61 -

*Alpinia galanga* (L.) Willd.  0.23 0.12 0.06 0.21

*Allium sativum* L. 0.23 0.01 0.01 0.01

*Cyperus rotundus* Linn. 0.23 0.12 0.06 0.10

*Glycyrrhiza glabra* Linn.  - 1.30 0.79 1.14

*Maerua siamensis* (Kurz) Pax.  0.23 0.12 0.06 0.10

*Piper retrofractum* Vahl. 0.23 0.12 0.06 0.10

*Phyllanthus emblica* L. 0.23 0.12 0.30 0.10

*Solanum torvum* Swartz. - 0.12 0.06 0.10

*Terminalia arjuna* Wight and Arn. 0.23 0.12 0.18 0.10

*Terminalia bellirica* (Gaertn.) Roxb. 0.23 0.12 0.18 0.10

*Terminalia citrina* Roxb. ex Fleming 0.23 0.12 0.06 0.10

*Tinospora crispa* (L.) Miers ex

Hook.f. & Thoms 0.23 0.01 0.01 0.01

*Zingiber officinale* Roscoe 0.23 0.12 0.06 0.31

The decoction yields were determined to be 14.56%, 22.20%, 8.00%, and 20.20 % (w/w) for Phy-Blica-O, Phy-Blica-B, Phy-Blica-D, and Phy-Blica-E, respectively.
