# Supplementary material for: Functional beverage development from traditional Thai polyherbal tonic: Antioxidant-rich microcapsules and comprehensive sub-chronic toxicity assessment
Source: PLoS One. 2025 Dec 23;20(12):e0339571. doi: 10.1371/journal.pone.0339571 (PMC12725736; doi:10.1371/journal.pone.0339571)
Supplement: S2 Table — (DOCX) [file pone.0339571.s002.docx]

**Supplementary Table S2** Sensory data for functional herbal tea based on *Phyllanthus emblica* with varying proportions of herbs and spices.

**Samples Appearance Odor Taste Overall acceptability**

Phy-Blica-O 5.27±0.34^a^ 5.53±0.30^a^ 4.63±0.32^b^ 5.90±0.31^ab^

Phy-Blica-B 7.00±0.28^a^ 6.70±0.29^a^ 6.20±0.29^a^ 6.77±0.23^a^

Phy-Blica-D 6.20±0.32^ab^ 6.57±0.27^a^ 6.27±0.27^a^ 6.40±0.23^a^

Phy-Blica-E 5.70±0.36^b^ 5.50±0.28^a^ 5.27±0.29^ab^ 5.23±0.23^b^

^a–d^Values are presented as mean±SD. Means in a row sharing a common superscript letter are not significantly different (*p*<0.05) as analyzed by One-way ANOVA followed by Bonferroni's post-hoc comparison tests.
