# Supplementary material for: Functional beverage development from traditional Thai polyherbal tonic: Antioxidant-rich microcapsules and comprehensive sub-chronic toxicity assessment
Source: PLoS One. 2025 Dec 23;20(12):e0339571. doi: 10.1371/journal.pone.0339571 (PMC12725736; doi:10.1371/journal.pone.0339571)
Supplement: S2 Fig — Particle sizes are displayed as differential volume (%) and cumulative volume (%) distribution curves. (DOCX) [file pone.0339571.s004.docx]

**
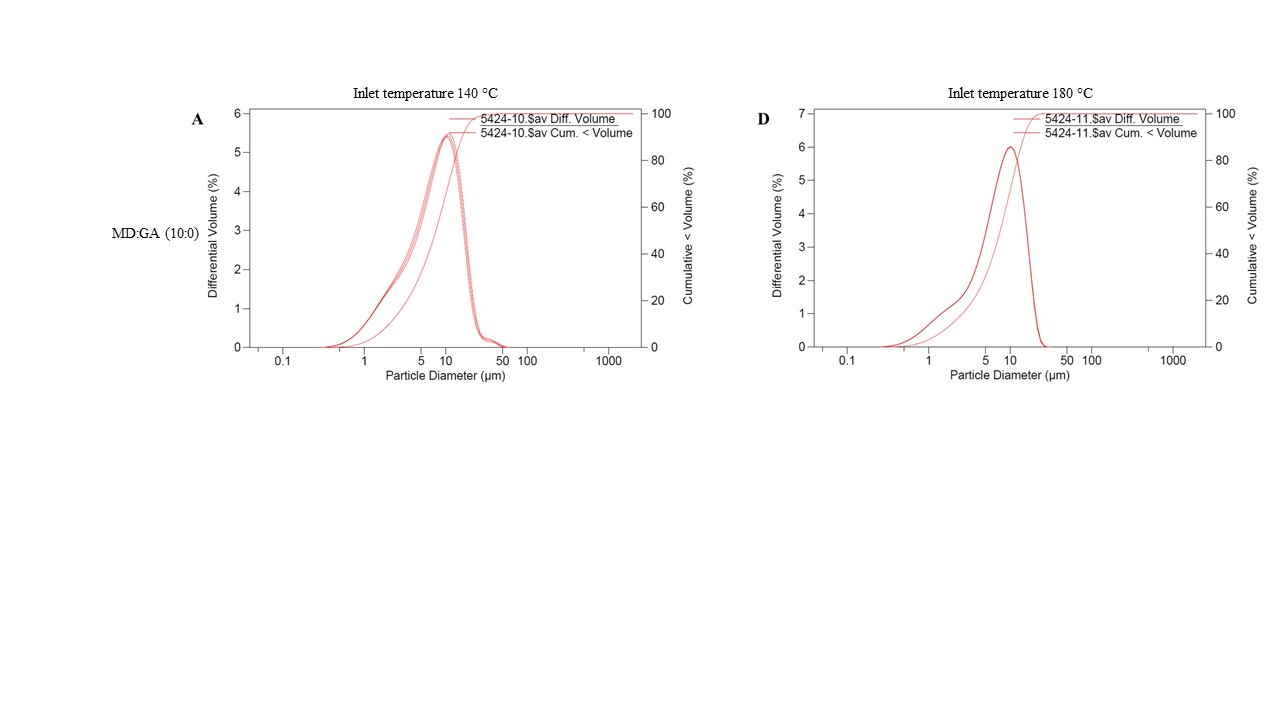

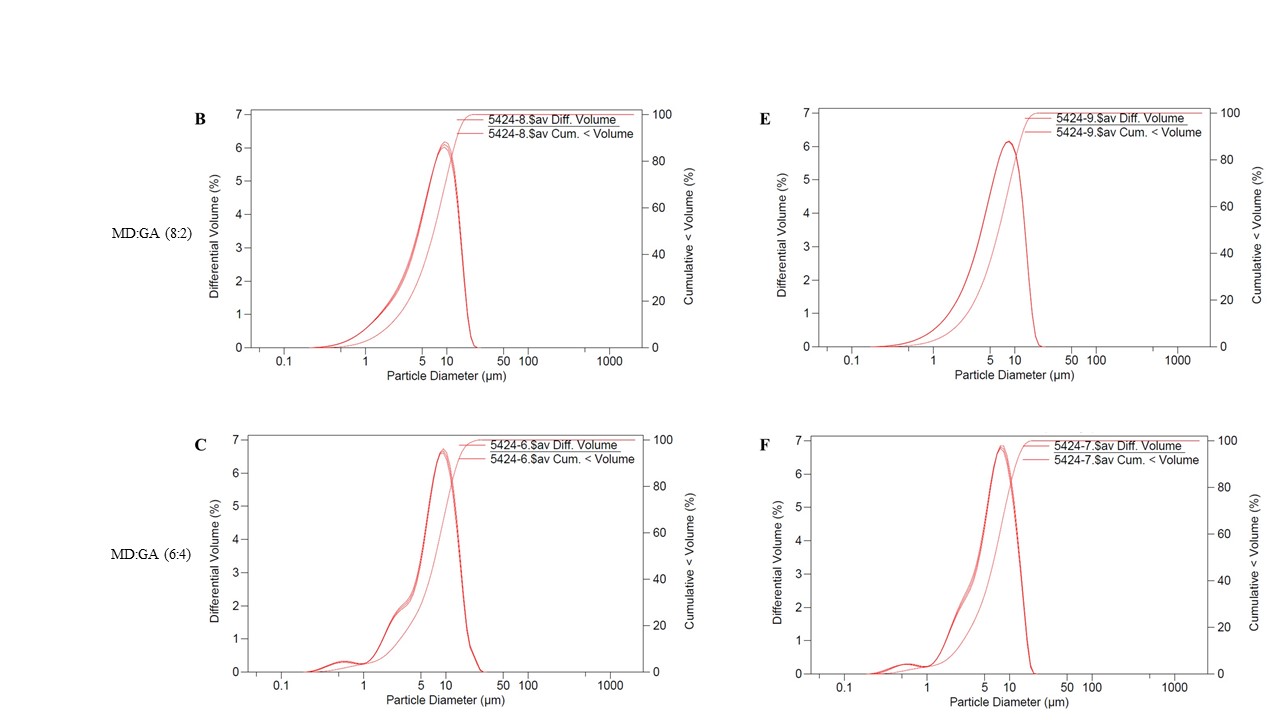
**

**Supplementary Fig. S2** The particle size distribution profiles of microencapsulated polyphenol-rich extracts from Phy-Blica-O powders were prepared by spray drying at various inlet temperatures (140°C: panels A–C; 180°C: panels D–F) and different maltodextrin (MD) to gum Arabic (GA) ratios: (A, D) MD:GA (10:0), (B, E) MD:GA (8:2), and (C, F) MD:GA (6:4). Particle sizes are displayed as differential volume (%) and cumulative volume (%) distribution curves.
