# Supplementary material for: Functional beverage development from traditional Thai polyherbal tonic: Antioxidant-rich microcapsules and comprehensive sub-chronic toxicity assessment
Source: PLoS One. 2025 Dec 23;20(12):e0339571. doi: 10.1371/journal.pone.0339571 (PMC12725736; doi:10.1371/journal.pone.0339571)
Supplement: S3 Fig — Fluorescence decay curves illustrate the relative fluorescence intensity (%) over 90 minutes in the presence of various sample concentrations (µg/mL): (A) Trolox (reference antioxidant), (B) Phy-Blica-O, (C) Phy-Blica-B, (D) Phy-Blica-D, and (E) Phy-Blica-E. A more significant inhibition of fluorescence decay indicates more potent antioxidant activity. (DOCX) [file pone.0339571.s005.docx]

**
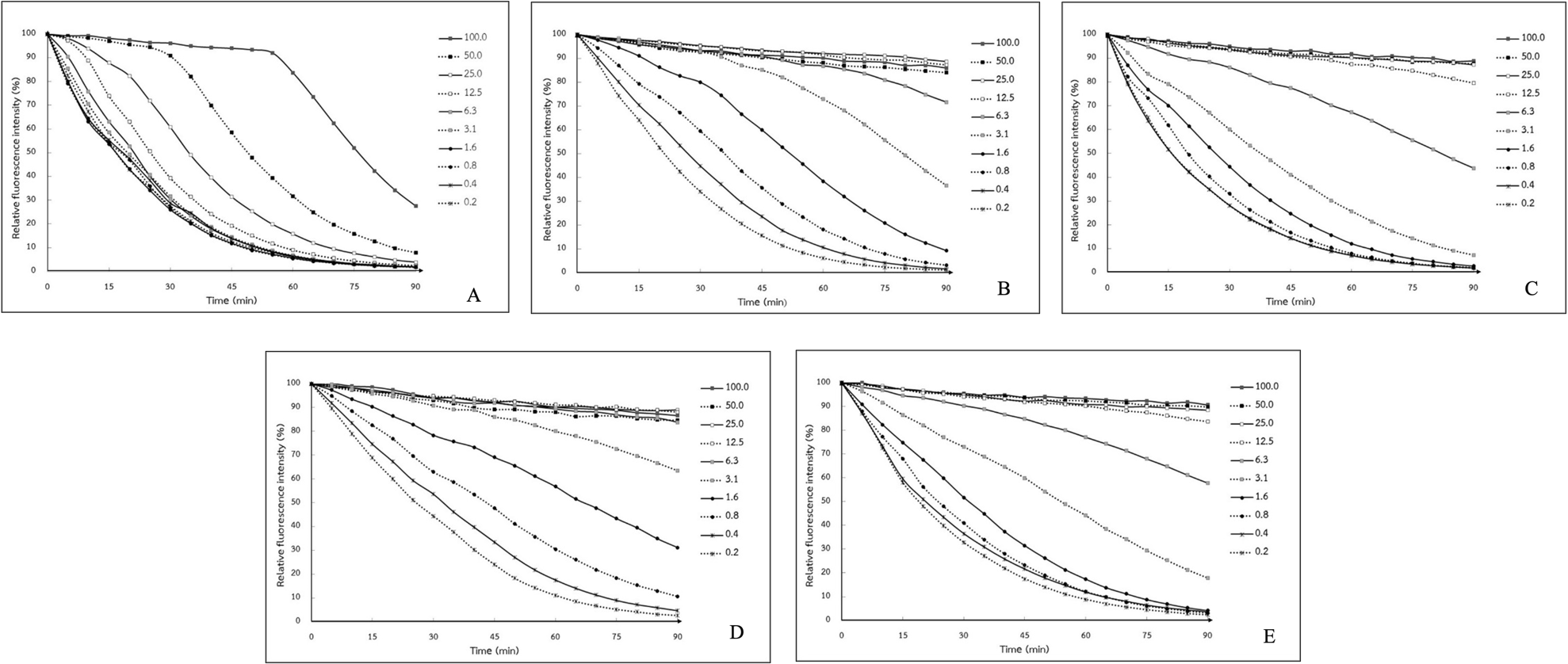
**

**Supplementary Fig. S3** The antioxidant activity of Phy-Blica herbal tea formulations is assessed by the inhibition of fluorescence decay in fluorescein. Fluorescence decay curves illustrate the relative fluorescence intensity (%) over 90 minutes in the presence of various sample concentrations (µg/mL): (A) Trolox (reference antioxidant), (B) Phy-Blica-O, (C) Phy-Blica-B, (D) Phy-Blica-D, and (E) Phy-Blica-E. A more significant inhibition of fluorescence decay indicates more potent antioxidant activity.
