# Supplementary material for: Functional beverage development from traditional Thai polyherbal tonic: Antioxidant-rich microcapsules and comprehensive sub-chronic toxicity assessment
Source: PLoS One. 2025 Dec 23;20(12):e0339571. doi: 10.1371/journal.pone.0339571 (PMC12725736; doi:10.1371/journal.pone.0339571)
Supplement: Attachment — Submitted filename: Respond to reviewer (point-by-point) Revised_(PBO and PBD).docx [file pone.0339571.s007.docx]

School of Health Science,

Mae Fah Luang University

14^th^ July 2025

Editor-in-Chief

PLOS ONE

Dear Editors,

On behalf of my co-authors, I am pleased to resubmit our revised manuscript titled: “Functional Beverage Development from Traditional Thai Polyherbal Tonic: Antioxidant-Rich Microcapsules and Comprehensive Sub-Chronic Toxicity Assessment” (Manuscript ID: PONE-D-25-18265) for reconsideration for publication in PLOS ONE. We would like to express our sincere gratitude to the editorial team and reviewers for their thoughtful and constructive feedback. We have thoroughly addressed all comments and suggestions in the revised manuscript and the accompanying point-by-point response document, as listed at the end of the cover letter.

Key revisions include (1) clarifying the study’s objectives, methods, major findings—including specific antioxidant assay results—and toxicological organ assessment, as well as correcting the HED expression and units. (2) Strengthening the introduction by emphasizing the importance of herbal beverages, the rationale for spray-drying, the role of microencapsulation (especially using maltodextrin and gum Arabic), and clearly identifying the research gap before outlining the study objectives. (3) Updating the methods section with complete equipment details (model, brand, country), justifying solvent and temperature choices, explaining the statistical tests for sensory data, and including animal ethics components such as anesthesia, sacrifice methods, and efforts to reduce suffering. (4) Expanding the results and discussion section with detailed statistical outcomes, clarifying the trade-offs between CE180_6:4 and CE140_6:4, discussing hygroscopicity and potential solutions, and adding sensory data and trends in toxicity over time. (5) Ensuring the conclusion is balanced and aligned with the study objectives, noting limitations such as storage stability and scalability, and providing clear recommendations for future research and practical applications.

We have also fully complied with PLOS ONE's journal-specific requirements, including reformatting the manuscript according to PLOS style templates and correcting the funding statement to accurately reflect the role of each funding source. Specifically: “This research was supported by Prince of Songkla University (Pattani campus) (Grant No. SAT601363S) for the in vivo experiment and encapsulation preparation, and was partially supported by Mae Fah Luang University (Grant No. 651C5003) for in vitro antioxidant testing. Mae Fah Luang University also covered the article processing fees. The funders had no role in study design, data collection and analysis, decision to publish, or manuscript preparation.” All funding-related information has been moved from the Acknowledgments section to the Funding Statement, as instructed. Supporting information files have been assigned appropriate figure and table captions and cited properly in the main text. All animal welfare guidelines have been updated to include methods of anesthesia, sacrifice, and measures to minimize suffering.

We believe that the revised version significantly improves the clarity, methodological transparency, and scientific rigor of our work, aligning with PLOS ONE’s editorial standards and scope. I appreciate your consideration and look forward to your favorable response.

Best Regards,

**Sasitorn Chusri, Ph.D.**
Corresponding Author
Biomedical Technology Research Group for Vulnerable Populations,
School of Health Science, Mae Fah Luang University
Muang, Chiang Rai 57100, Thailand
E-mail: [sasitorn.chu@mfu.ac.th](mailto:sasitorn.chu@mfu.ac.th)

**Journal Requirements:**

When submitting your revision, we need you to address these additional requirements.

1. Please ensure that your manuscript meets PLOS ONE's style requirements, including those for file naming. The PLOS ONE style templates can be found at https://journals.plos.org/plosone/s/file?id=wjVg/PLOSOne_formatting_sample_main_body.pdf and <https://journals.plos.org/plosone/s/file?id=ba62/PLOSOne_formatting_sample_title_authors_affiliations.pdf>

**Author Response:** The format has been checked and corrected as suggested.

2. To comply with PLOS ONE submissions requirements, in your Methods section, please provide additional information regarding the experiments involving animals and ensure you have included details on (1) methods of sacrifice, (2) methods of anesthesia and/or analgesia, and (3) efforts to alleviate suffering.

**Author Response**: The information has been updated in the Methods section.

3. Please note that PLOS ONE has specific guidelines on code sharing for submissions in which author-generated code underpins the findings in the manuscript. In these cases, we expect all author-generated code to be made available without restrictions upon publication of the work. Please review our guidelines at https://journals.plos.org/plosone/s/materials-and-software-sharing#loc-sharing-code and ensure that your code is shared in a way that follows best practice and facilitates reproducibility and reuse.

**Author Response**: We noted the mentioned information.

4. We note that the grant information you provided in the ‘Funding Information’ and ‘Financial Disclosure’ sections do not match. When you resubmit, please ensure that you provide the correct grant numbers for the awards you received for your study in the ‘Funding Information’ section.

**Author Response**: The information has been corrected in the revised version.

5. Thank you for stating the following financial disclosure:

This research was supported by the Prince of Songkla University (Pattani campus) (Grant No. SAT601363S) and was partially supported by Mae Fah Luang University (Grant No. 651C5003). Mae Fah Luang University covered the article processing fees charge.

Please state what role the funders took in the study. If the funders had no role, please state: "The funders had no role in study design, data collection and analysis, decision to publish, or preparation of the manuscript." If this statement is not correct you must amend it as needed.

Please include this amended Role of Funder statement in your cover letter; we will change the online submission form on your behalf.

**Author Response**: The roles of each funding were assigned as follows. This research was supported by Prince of Songkla University (Pattani campus) (Grant No. SAT601363S) for the *in vivo* experiment and encapsulation preparation, and it was partially funded by Mae Fah Luang University (Grant No. 651C5003) for conducting *in vitro* antioxidant activities. Mae Fah Luang University also covered the article processing fees.

6. Thank you for stating the following in the Acknowledgments Section of your manuscript:

This research was supported by the Prince of Songkla University (Pattani campus) (Grant No. SAT601363S) and was partially supported by Mae Fah Luang University (Grant No. 651C5003). Mae Fah Luang University covered the article processing fees charge. We note that you have provided funding information that is not currently declared in your Funding Statement. However, funding information should not appear in the Acknowledgments section or other areas of your manuscript. We will only publish funding information present in the Funding Statement section of the online submission form. Please remove any funding-related text from the manuscript and let us know how you would like to update your Funding Statement. Currently, your Funding Statement reads as follows: This research was supported by the Prince of Songkla University (Pattani campus) (Grant No. SAT601363S) and was partially supported by Mae Fah Luang University (Grant No. 651C5003). Mae Fah Luang University covered the article processing fees charge. Please include your amended statements within your cover letter; we will change the online submission form on your behalf.

**Author Response**: The roles of each funding were assigned as follows. This research was supported by Prince of Songkla University (Pattani campus) (Grant No. SAT601363S) for the *in vivo* experiment and encapsulation preparation, and it was partially funded by Mae Fah Luang University (Grant No. 651C5003) for conducting *in vitro* antioxidant activities. Mae Fah Luang University also covered the article processing fees.

7. Your ethics statement should only appear in the Methods section of your manuscript. If your ethics statement is written in any section besides the Methods, please delete it from any other section.

**Author Response**: The information has been corrected in the revised version.

8. Please include captions for your Supporting Information files at the end of your manuscript, and update any in-text citations to match accordingly. Please see our Supporting Information guidelines for more information: http://journals.plos.org/plosone/s/supporting-information.

**Author Response**: The information has been corrected in the revised version.

**Reviewer #1: PLOS ONE**

Functional beverage development from traditional polyherbal tonic: Antioxidant-rich microcapsules and comprehensive sub-chronic toxicity assessment”

General comment: The manuscript in reference compiles the reported “Functional beverage development from traditional polyherbal tonic: Antioxidant-rich microcapsules and comprehensive sub-chronic toxicity assessment”. The manuscript has relevant information and organization that will be interesting for readers. However, some points need to be addressed before further consideration.

1. Abstract: The writing style should be concise, clear, and reflect the objectives, research methods, results, conclusions, and suggestions in full.

**Author Response:** We sincerely thank the reviewer for their valuable suggestion. In response, we have revised the abstract to clearly and concisely present the study’s objective, methodology, key findings, conclusion, and potential applications in the revised version.

2. Introduction: -What are the highlights of this article? Should add details.

**Author Response:** Thank you for this insightful suggestion. We have revised the Introduction section to clearly highlight the main points and importance of the study. Specifically, we have (1) clarified the reason for transforming Phy-Blica-O (a traditional polyherbal tonic) into a functional ingredient through microencapsulation, (2) emphasized the novelty of combining optimization of spray-drying conditions with extensive physicochemical and antioxidant assessments, (3) highlighted the scientific and regulatory importance of conducting a 90-day sub-chronic oral toxicity study to ensure safety for human use, and (4) stressed the translational potential of PBD as a safe, antioxidant-rich functional beverage suitable for use in complementary and integrative medicine.

3. Method:

-Should be shown a flow chart as an illustration of 2.1, 2.2, and 2.3.

-Should be added details of equipment in this study, including model, brand, and country of manufacture.

**Author Response:** We appreciate the reviewer for the helpful suggestion. In response, we have added a flow chart, now labeled as Figure 1, to visually illustrate the experimental workflow described in Sections 2.1 (Preparation of Phy-Blica-O herbal tea), 2.2 (Development of decoction and phenolic-rich extracts), and 2.3 (Microencapsulation via spray-drying). This illustration is intended to enhance reader comprehension of the stepwise procedures and clarify the overall methodology.

Furthermore, we have thoroughly revised the Methods section to include the full specifications of all major equipment used in the study. This consists of the model number, brand name, and country of manufacture for equipment involved in freeze drying, spray-drying, particle analysis, microscopy, colorimetry, moisture, and water activity measurements.

4. Discussion:
- “Optimal encapsulation efficiencies for the PBO and its phenolic-rich extracts were achieved at lower drying temperatures (140°C), with higher proportions of MD, consistent with previous research highlighting the effectiveness of maltodextrin in preserving bioactive components during drying processes [26-28].” Should be explained the role of maltodextrin in preserving bioactive components during the drying process.

**Author Response:** We thank the reviewer for this helpful suggestion. In response, we have expanded the relevant sentence in the Discussion section to clarify the mechanistic role of maltodextrin (MD) in preserving bioactive compounds during spray-drying as follows.

“Optimal encapsulation efficiencies for the PBO and its phenolic-rich extracts were achieved at lower drying temperatures (140°C), with higher proportions of MD, consistent with previous research highlighting the effectiveness of maltodextrin in preserving bioactive components during drying processes [26-28]. This protective role is attributed to its low viscosity, high water solubility, and excellent film-forming ability, which contribute to forming a stable encapsulating matrix around heat-sensitive phytochemicals, thereby minimizing thermal degradation and oxidation during spray-drying [26–28].”

-“Nevertheless, formulations with increased GA content exhibited elevated
hygroscopicity, a characteristic previously associated with GA, underscoring the necessity of selecting appropriate encapsulation materials [34, 35].” It should be clearly explained, including examples of related research and its relationship to the results obtained in this research.

**Author Response:** We appreciate the reviewer’s insightful suggestion. In response, we have revised the discussion to explain the hygroscopic properties of gum Arabic better, supported by literature and linked to our findings as follows.

“Nevertheless, formulations with increased GA content exhibited elevated hygroscopicity, a characteristic previously associated with gum Arabic due to its high affinity for moisture and its molecular structure rich in hydrophilic polysaccharides. This property causes microcapsules containing higher GA proportions to absorb more atmospheric water during storage, potentially compromising their stability and shelf life. For example, Ahmadi et al. (2025) demonstrated that clove extract microcapsules containing higher ratios of GA showed significantly greater moisture uptake after 7 days compared to those encapsulated with maltodextrin alone [34]. Similarly, Akhavan Mahdavi et al. (2016) reported that gum Arabic increased water sorption and reduced flowability in anthocyanin powders [35]. These findings are consistent with our results, where microcapsules with a 6:4 MD:GA ratio (e.g., CE180_6:4) showed the highest hygroscopicity (15.51% after 7 days), reinforcing the need to optimize wall material composition to balance bioactive preservation and physical stability.”

5. Conclusion:

-Should be rewritten and summarized important points that consistent with the article objectives

-Should be added suggestions, utilization, and guidelines for future development.

**Author Response:** We thank the reviewer for this valuable recommendation. In response, we have revised the Conclusion section to better reflect the core objectives of the study, which are (i) optimizing microencapsulation of Phy-Blica-O (PBO) and (ii) evaluating the antioxidant capacity and sub-chronic toxicity of Phy-Blica-D (PBD), as well as incorporating recommendations for future research, potential applications, and product development guidelines as follows.

“This study successfully developed and optimized PBO into a functional ingredient using spray-drying microencapsulation. Optimal encapsulation efficiency was achieved at 140°C with maltodextrin as the wall material. At the same time, superior antioxidant activity was observed in formulations prepared at 180°C with a maltodextrin:gum Arabic ratio of 6:4. These encapsulated powders demonstrated favorable physicochemical properties, which may contribute to improve their stability and bioactivity. The resulting formulation, PBD, showed high consumer acceptability, potent in vitro antioxidant activity, and no observed adverse effects in a 90-day sub-chronic oral toxicity study, with a NOAEL exceeding 300 mg/kg/day. These results confirm the potential of PBD as a safe, antioxidant-rich functional beverage. For practical utilization, this research supports developing PBD as a commercially viable product within the functional food and complementary medicine industries. The encapsulated powder could be incorporated into ready-to-drink teas, dietary supplements, or wellness formulations targeting oxidative stress-related health concerns. However, further evaluation of the long-term storage stability of microencapsulated powders under real-world conditions, along with the bioavailability and pharmacokinetics of the key bioactive compounds *in vivo*, is necessary for manufacturing and application.”

6. Other Suggestions:

-The reference should be checked according to the journal's requirements.

-Should be updated references.

**Author Response:** We sincerely thank the reviewer for this important comment. In response:

- Reference Format: All references have been carefully reviewed and reformatted to fully comply with PLOS ONE's citation style, which follows the Vancouver style with the following structure: Author(s). Title. Journal Abbreviation. Year;Volume(Issue):Page range. doi (if available).
- Updated References: We have updated and replaced outdated or superseded citations with more recent publications (mostly from the past 5 years). This ensures the manuscript reflects the current state of research in functional food development, microencapsulation, and polyherbal safety assessment.
- DOI Inclusion: Where applicable, DOIs have been added to all journal articles, in accordance with PLOS ONE’s submission guidelines. We believe these adjustments improve both the academic rigor and alignment with the journal’s formatting standards.

**Reviewer #2:** The manuscript presents a comprehensive study on the development of a functional beverage, Phy-Blica-D (PBD), derived from a traditional Thai polyherbal tonic, Phy-Blica-O (PBO), through microencapsulation and sub-chronic toxicity assessment. However, the manuscript has some limitations, including incomplete statistical reporting, limited discussion of encapsulation trade-offs, and minor inconsistencies in terminology and data presentation. With revisions, the manuscript could make a significant contribution to the field of functional beverages and complementary medicine:

1. Title: Revise the short title to correct the grammatical error: “Phy-Blica-O and Phy-Blica-D: Development of Microcapsules and Sub-Chronic Toxicity Assessment.” Consider specifying “Thai” in the main title for clarity: “Functional Beverage Development from Traditional Thai Polyherbal Tonic...” Ensure keyword consistency (e.g., capitalize “Sub-Chronic Toxicity” to match others).

**Author Response:** We appreciate the reviewer’s detailed and helpful suggestions. For the Short Title, we have revised it to correct the grammatical error and improve clarity and conciseness. The original “Phy-Blica-O and Phy-Blica-D: Development of Microcapsules and Sub-Chronic Toxicity Assessment” has been updated to "Development of Microcapsules and Sub-Chronic Toxicity of Phy-Blica Formulations."

Furthermore, we agree with the suggestion to clarify the geographic and traditional context by specifying "Thai" in the main title. Therefore, the main title was changed from ‘Functional Beverage Development from Traditional Polyherbal Tonic: Antioxidant-Rich Microcapsules and Comprehensive Sub-Chronic Toxicity Assessment’ to ‘Functional Beverage Development from Traditional Thai Polyherbal Tonic: Antioxidant-Rich Microcapsules and Comprehensive Sub-Chronic Toxicity Assessment’

We have ensured consistency by capitalizing "Sub-Chronic Toxicity" to align with the formatting of other keywords. We believe these revisions enhance the clarity, specificity, and consistency of the manuscript presentation and are now fully aligned with journal expectations.

2. Abstract: Clarify the antioxidant activity claim by specifying which assays showed superiority for CE180_6:4 or acknowledge variability across assays. Correct or explain the HED calculation to ensure unit consistency (e.g., convert to volume based on beverage concentration). Specify the organs examined in the toxicity study for transparency.

**Author Response:** We sincerely thank the reviewer for this thoughtful and constructive feedback. In response, we have revised the Abstract to address all points raised:

- Antioxidant Activity of CE180_6:4: We have clarified that the CE180_6:4 microcapsule formulation showed superior antioxidant activity based on specific in vitro assays. The revised sentence now states:“Maximum encapsulation efficiency was achieved at 140°C with MD-only wall materials, while microcapsules with a 6:4 MD:GA ratio at 180°C exhibited the highest antioxidant activity based on DPPH, ABTS, and FRAP assays.” This provides clear attribution of assay-specific performance rather than implying universal superiority.
- Human Equivalent Dose (HED): To ensure unit consistency, we have clarified how the HED was derived from the NOAEL and converted to an approximate beverage volume based on the concentration used in the study. The revised sentence reads: “The calculated human equivalent dose is 50.27 mg/kg/day, which corresponds to an estimated volume of approximately 600 mL/kg/day based on the extraction yield obtained from the beverage.”
- Organs Examined in Toxicity Study: We have added detail on the organs examined for histopathological analysis to improve transparency. The sentence now reads: “The oral no-observed-adverse-effect level (NOAEL) exceeded 300 mg/kg/day, with normal histopathological profiles in the liver, kidneys, and spleen.”

3. Introduction: Streamline the functional food discussion to focus on herbal beverages and their specific benefits. Justify the choice of spray-drying by briefly comparing it to other encapsulation methods or citing its advantages (e.g., scalability, cost-effectiveness). Clarify the cytotoxicity findings (e.g., specify concentrations tested) to strengthen the safety claim. There is no explanation why encapsulation is important for PBO and PBD. There is no explanation why GA and MD were used for encapsulation of PBO and PBD. Please explain in details the gap in the research before the objectives.

**Author Response:** We thank the reviewer for these insightful comments. In response, we have carefully revised the Introduction section to address each point and strengthen the scientific rationale of the study. The following amendments have been made:

- Streamlining the functional food discussion: We revised the beginning of the introduction to specifically highlight herbal beverages as a subgroup of functional foods. The updated paragraph emphasizes their contribution to health, antioxidant properties, and traditional uses, rather than broadly discussing all types of functional foods.
- Justification for spray-drying: We have now included a brief comparison of encapsulation methods and provided the rationale for using spray-drying, with relevant citations added to support this justification.
- Clarification of cytotoxicity findings: We have expanded the discussion of our previous cytotoxicity work by stating ‘Among these formulas, *Phyllanthus emblica*-based functional herbal tea (THP-R016 or Phy-Blica-O (PBO)) possessed notable antioxidant properties and did not exhibit cytotoxic effects on *Vero* cells at concentrations up to 100 µg/mL, supporting its safety for oral applications [12, 13].’
- Importance of encapsulation for PBO and PBD: We have added a paragraph to explain why encapsulation is critical, stating that: ‘The utilization of microencapsulation in PBO not only enhances the sensory profile but also protects heat-sensitive bioactive compounds from degradation, thereby improving the functional properties and shelf-life of the formulation [14].’
- Rationale for using gum Arabic (GA) and maltodextrin (MD): We have now provided a detailed rationale, as follows. ‘Additionally, maltodextrin (MD) and gum Arabic (GA) are common wall materials in spray-drying botanical extracts because of their excellent film-forming, emulsifying, and protective qualities [15-17]. MD helps achieve high encapsulation efficiency and maintains low viscosity, whereas GA improves stability and retains antioxidants. Using them together has been demonstrated to increase encapsulation yields and functional performance in earlier research [15–17].’

4. Materials and methods: Justify the solvent choices and pH adjustments for phenolic extraction, citing relevant literature or preliminary experiments. Explain the selection of inlet temperatures (140°C, 180°C) based on prior studies or thermal stability of PBO components. What about the single treatment of MD or GA not in a combination. Clarify the statistical analysis for sensory data (e.g., ANOVA, significance level) and report results in a table or supplementary file. Include weekly body weight and food consumption trends in the toxicity study (e.g., as a supplementary figure) to show temporal patterns.

**Author Response:** We appreciate the reviewer’s detailed and insightful comments. In response, we have revised the Materials and Methods and Results section as follows:

- Justification of solvent and pH adjustments in phenolic extraction: We have added a justification for using diethyl ether, chloroform, and ethyl acetate in the sequential extraction of phenolic-rich fractions from PBO, with pH adjustments to target selective partitioning of non-polar and polar phenolics. Specifically, we now state ‘The stepwise solvent extraction method employed here follows established protocols for phenolic isolation [20, 21], where initial basification (pH 8–10) enhances solubility of non-phenolics, while subsequent acidification (pH 3–4) facilitates phenolic partitioning into ethyl acetate. This approach was validated in prior studies [20, 21] for optimizing polyphenol yield from botanical extracts.’
- Rationale for inlet temperatures (140°C and 180°C): We have clarified that the selected inlet temperatures were based on prior encapsulation studies of plant-based extracts, balancing drying efficiency and thermal degradation risk. ‘Inlet air temperatures were set at 140 and 180 °C, which were chosen based on previous studies on spray-drying of herbal extracts [16, 20-21], with outlet temperatures recorded for each sample.’
- Single treatment of MD or GA: We have explained that both individual and combined wall materials were tested to explore their individual effects and synergistic roles in encapsulation.
- Statistical analysis of sensory data: We have clarified the statistical approach for the sensory evaluation, including significance testing and reporting format. The revised text states: ‘Sensory scores and group differences were examined using one-way ANOVA with Bonferroni’s post-hoc tests’ in the methods section. Furthermore, the revised text was added to the results section: ‘To enhance consumer acceptance, we incorporated various edible medicinal plants and spices into the formulation. **Supplementary Table 2** presents the sensory properties of *P. emblica*-based herbal tea samples made with different medicinal plants.’
- Weekly body weight and food consumption trends: To illustrate temporal trends in the 90-day sub-chronic toxicity study, we have now included weekly body weight and food consumption data in Figure 4. This allows readers to assess consistency and potential treatment-related deviations over time.

5. Results and discussion: Provide detailed statistical results (e.g., p-values, effect sizes) for antioxidant and toxicity data, and discuss significant comparisons in the text. Acknowledge the trade-off between TPC (higher in CE140_6:4) and other antioxidant assays (higher in CE180_6:4), and explain why CE180_6:4 was deemed optimal. Discuss potential solutions for high hygroscopicity (e.g., modified packaging, lower GA ratios) to enhance commercial viability. Include a table or figure summarizing sensory scores and panelist preferences to support claims about PBD’s consumer acceptability. Clarify the HED calculation, specifying the beverage concentration (e.g., mg/mL) and correcting the volume-based expression (mL/kg).

Author Response: We sincerely thank the reviewer for the detailed and constructive feedback. In response, we have revised the Results and Discussion sections to address each point, as outlined below:

- Statistical Reporting of Antioxidant and Toxicity Data: We have rechecked and updated the Results section, particularly Tables and Figures, to report specific p-values for key comparisons in antioxidant assays (DPPH, ABTS, FRAP) and toxicity outcomes (e.g., organ weights, biochemical markers). Statistical significance was indicated using superscript letters in tables, and results were described explicitly in the text.
- Interpretation of TPC vs. Antioxidant Activity (CE140_6:4 vs. CE180_6:4): We have acknowledged and discussed the trade-off in the antioxidant data in the results section as follow: “Although CE140_6:4 exhibited the highest TPC, CE180_6:4 consistently showed superior activity in DPPH, ABTS, and FRAP assays (p < 0.05). This suggests that antioxidant potency may be driven not only by TPC quantity but also by the retention and stability of specific phenolic compounds during high-temperature drying. Collectively, CE180_6:4 consistently showed superior antioxidant performance across multiple antioxidant assays, marking it as the optimal microcapsule formulation for maximizing antioxidant activity.”
- Discussion of High Hygroscopicity and Commercial Solutions: We have expanded the discussion on the commercial limitations of high GA content formulations in the discussion section as follows: ‘Furthermore, this study did not thoroughly evaluate the long-term stability of the encapsulated herbal powder under actual commercial storage conditions, which is crucial for establishing practical shelf-life guidelines. Significantly, the increased hygroscopicity seen in CE180_6:4 (15.51% after 7 days) poses a storage challenge. To address this, future formulations might lower the GA proportion or add encapsulation stabilizers. Additionally, enhancing commercial viability could involve moisture-barrier packaging (such as aluminum foil laminates with desiccants) and cold-chain storage systems.’
- Sensory Score Summary: We have included Supplementary Table S2 summarizing mean sensory scores (taste, aroma, appearance, overall acceptability) and panelist preference rankings in the results as follows: ‘It should be noted that among the five formulations tested, Phy-Blica-D (PBD) scored the highest in taste (5.50±0.35; p<0.05) and overall acceptability (5.60±0.37; p<0.05), significantly outperforming PBO (2.70±0.34 and 2.90±0.36).
- Clarification of Human Equivalent Dose (HED): We have corrected and clarified the HED expression to ensure unit consistency. The revised text in the results section is as follows: ‘The NOAEL of 300 mg/kg/day in rats corresponds to a human equivalent dose (HED) of 50.27 mg/kg/day based on standard conversion [50]. Given that the extract concentration in PBD was approximately 83.8 mg/100 mL (0.838 mg/mL), this HED equates to an approximate beverage intake of 600 mL/kg/day.’

6. Conclusions: Balance the claim about CE180_6:4 by noting the TPC advantage of CE140_6:4 and clarifying the basis for optimality. Correct the HED expression and provide the beverage concentration used for the volume estimate. Expand the limitations to include stability testing and scalability challenges, with suggestions for future research.

**Author Response:** We thank the reviewer for these important suggestions. In response, we have revised the Conclusion section to ensure a more balanced interpretation of the findings, correct dosing calculations, and expand the study limitations. The following changes have been made:

- Balanced Interpretation of CE180_6:4 vs. CE140_6:4: We have acknowledged the higher total phenolic content (TPC) in CE140_6:4 and clarified why CE180_6:4 was considered optimal in the discussion section as follows: ‘“Although CE140_6:4 exhibited the highest TPC, CE180_6:4 consistently showed superior activity in DPPH, ABTS, and FRAP assays (p < 0.05). This suggests that antioxidant potency may be driven not only by TPC quantity but also by the retention and stability of specific phenolic compounds during high-temperature drying. Collectively, CE180_6:4 consistently showed superior antioxidant performance across multiple antioxidant assays, marking it as the optimal microcapsule formulation for maximizing antioxidant activity.”
- Corrected HED Expression and Beverage Concentration: We have corrected the HED statement for clarity and unit consistency. The revised text in the results section is as follows: ‘The NOAEL of 300 mg/kg/day in rats corresponds to a human equivalent dose (HED) of 50.27 mg/kg/day based on standard conversion [50]. Given that the extract concentration in PBD was approximately 83.8 mg/100 mL (0.838 mg/mL), this HED equates to an approximate beverage intake of 600 mL/kg/day.’
- Expanded Limitations and Future Research Directions: We have expanded the limitations paragraph to include concerns about storage stability in the discussion section as follows: ‘Furthermore, this study did not thoroughly evaluate the long-term stability of the encapsulated herbal powder under actual commercial storage conditions, which is crucial for establishing practical shelf-life guidelines. Significantly, the increased hygroscopicity seen in CE180_6:4 (15.51% after 7 days) poses a storage challenge. To address this, future formulations might lower the GA proportion or add encapsulation stabilizers. Additionally, enhancing commercial viability could involve moisture-barrier packaging (such as aluminum foil laminates with desiccants) and cold-chain storage systems.’
