# Supplementary material for: Functional beverage development from traditional Thai polyherbal tonic: Antioxidant-rich microcapsules and comprehensive sub-chronic toxicity assessment
Source: PLoS One. 2025 Dec 23;20(12):e0339571. doi: 10.1371/journal.pone.0339571 (PMC12725736; doi:10.1371/journal.pone.0339571)
Supplement: Attachment — Submitted filename: (PBO and PBD)_Reponse to reviewer.docx [file pone.0339571.s008.docx]

School of Health Science,

Mae Fah Luang University

30^th^ November 2025

Editor-in-Chief

PLOS ONE

Subject: Submission of Manuscript titled “Functional beverage development from traditional polyherbal tonic: Antioxidant-rich microcapsules and comprehensive sub-chronic toxicity assessment.”

Dear Editors,

On behalf of my co-authors, I am pleased to resubmit our revised manuscript titled: “Functional Beverage Development from Traditional Thai Polyherbal Tonic: Antioxidant-Rich Microcapsules and Comprehensive Sub-Chronic Toxicity Assessment” (Manuscript ID: PONE-D-25-18265R2) for reconsideration for publication in PLOS ONE. We would like to express our sincere gratitude to the editorial team and reviewers for their thoughtful and constructive feedback. We have thoroughly addressed all comments and suggestions in the revised manuscript and the accompanying point-by-point response document, as listed at the end of the cover letter.

We believe that the revised version significantly improves the clarity, methodological transparency, and scientific rigor of our work, aligning with PLOS ONE’s editorial standards and scope. I appreciate your consideration and look forward to your favorable response.

Best Regards,

**Sasitorn Chusri, Ph.D.**
Corresponding Author
Biomedical Technology Research Group for Vulnerable Populations,
School of Health Science, Mae Fah Luang University
Muang, Chiang Rai 57100, Thailand
E-mail: sasitorn.chu@mfu.ac.th

**Reviewer #2: Major Comments**

1. Statistical Reporting – While p-values and comparisons are included, effect sizes and confidence intervals should be more explicitly presented to strengthen result interpretation.

**Address to the reviewer’s comment:** We thank you for this helpful suggestion. In response, we've revised the Results section to explicitly report effect sizes with 95% confidence intervals where relevant. These updates help readers understand both the magnitude and precision of the effects, going beyond just p-values. Additionally, we've updated tables to show effect sizes alongside statistical results.

2. Encapsulation Trade-offs – The discussion should better highlight the balance between high TPC (CE140_6:4) and superior antioxidant activity (CE180_6:4), clarifying the rationale for selecting the “optimal” formulation.

**Address to the reviewer’s comment:** Thank you for this insightful comment. We appreciate the reviewer’s suggestion to more clearly explain the trade-offs between total phenolic content (TPC) and antioxidant activity and to clarify the rationale behind selecting the optimal formulation.

In response, we have revised the Results and Discussion to explain how these factors influenced the formulation choice explicitly. Specifically, we now note that although CE140_6:4 showed the highest TPC (102.05 mg GA/g extract), this did not necessarily indicate the best antioxidant performance. Instead, microcapsules produced at 180°C with a 6:4 MD:GA ratio (CE180_6:4) consistently exhibited stronger antioxidant activity in DPPH, ABTS, and FRAP assays, indicating better retention and stability of key bioactive compounds at higher drying temperatures. This suggests that antioxidant strength depends not only on the amount of phenolics but also on their chemical stability after encapsulation. This information was added to the results and discussion sections.

3. Hygroscopicity Limitation – The practical challenges of high GA content (storage instability) should be more explicitly connected to commercial implications and possible solutions.

**Address to the reviewer’s comment:** Thank you for your insightful comment. We agree that the practical impact of high hygroscopicity in GA-rich formulations should be more clearly linked to commercial considerations and potential mitigation strategies. Therefore, we expanded the discussion to emphasize how high GA levels, especially in formulations like CE180_6:4, which had the highest hygroscopicity (15.51% after 7 days), present real challenges for product stability during storage, handling, and distribution. The updated text now underscores that excessive moisture uptake can lead to particle caking, decreased flowability, reduced antioxidant effectiveness, and shorter shelf life, which are key factors for the commercial application of microencapsulated herbal ingredients. These modifications enhance the Discussion section by clearly connecting scientific results to practical formulation challenges and industry solutions.

To address these challenges, we included clearer solutions such as

(1) decreasing GA content or adjusting MD: GA ratios to balance phenolic protection and physical stability

(2) incorporating stabilizing co-encapsulants

(3) using moisture-barrier packaging methods (e.g., aluminum foil laminates, desiccants, low-permeability films).

4. Toxicity Study Reporting – Weekly body weight and food intake trends are added, but a brief summary of variability and overall trajectory in the main text would enhance clarity.

**Address to the reviewer’s comment:** Thank you for your helpful comment. We agree that summarizing the weekly body weight and food-intake trends in the main text would enhance clarity and readability. Therefore, we have expanded the toxicity Results section to include a brief narrative that emphasizes the overall variability and trajectory observed over the 90-day study. The revised text now indicates that both male and female rats in all treatment groups experienced consistent, gradual increases in body weight over 90 days, without any abnormal fluctuations or deviations from the control group. Weekly food intake patterns remained stable and similar across all groups, with only minor weekly variations typical of normal physiological cycles. Notably, no dose-dependent effects were observed in either parameter, reinforcing the absence of treatment-related toxicity. This summary improves the clarity of the weekly data while remaining aligned with the detailed figures provided.

Minor Comments
5. Abstract: Slightly long—could be streamlined for clarity, focusing on objective, key findings, and implications.

**Address to the reviewer’s comment:** Thank you for this constructive comment. We appreciate the suggestion to make the abstract clearer and more focused. In response, we have revised the abstract to be more concise, highlighting the study’s main goal, the key findings from microencapsulation optimization and toxicity testing, and the main implications for developing functional beverages.

6. Introduction: Nicely revised, though still somewhat broad. Consider reducing general functional food market data and focusing more directly on herbal beverages.

**Address to the reviewer’s comment:** Thank you for the insightful comment. We agree that the Introduction section would benefit from a tighter focus on herbal beverages rather than a broader discussion of the functional food market. We removed several sentences and citations related to overall trends in the functional food market. This revision offers a clearer context for the study by directly linking PBO and its microencapsulation to current research and development in herbal-based functional drinks.

7. Figures/Tables: Supplementary figures and tables are well presented. Ensure that all abbreviations are defined at first mention.

**Address to the reviewer’s comment:** Thank you for your positive feedback on the supplementary figures and tables. We appreciate the reviewer’s reminder regarding the clarity of abbreviations. In response, we reviewed the entire manuscript, including the supplementary figures, tables, and captions, to ensure that all abbreviations are defined at first use, covering methodological terms, assays, sample codes, and parameters. These updates improve clarity and consistency.

8. References: Updated references are appropriate, but a few older citations remain; consider substituting with more recent (past 5 years) where possible.

**Address to the reviewer’s comment:** Thank you for highlighting the importance of the updated references. We reviewed the reference list and replaced many older citations with recent studies (from the past 5 years), especially in areas like functional herbal beverages, microencapsulation, and antioxidant assessment. We retained a few older references that are still seminal or foundational for traditional Thai polyherbal formulations and microencapsulation methodology, where no recent alternatives exist. This blend of updated and classic references offers a current yet historically grounded context for the work.

9. Language: Overall clear, but some sentences could be shortened to improve readability.

**Address to the reviewer’s comment:** Thank you for this helpful comment. We have carefully re-read the entire manuscript, focusing on sentence length and readability. In response, we shortened several long, multi‑clause sentences, especially in the Introduction, Discussion, and Methods sections, and removed minor redundancies to improve flow without changing the scientific meaning.
